# Effect of COVID-19 on cardiorenal axis: known or unknown universe?

**DOI:** 10.1590/1414-431X2022e11932

**Published:** 2022-03-21

**Authors:** G.M. Armentano, M.S. Carneiro-Ramos

**Affiliations:** 1 Laboratório de Imunologia Cardiovascular, Centro de Ciências Naturais e Humanas, Universidade Federal do ABC, Santo André, SP, Brasil

**Keywords:** COVID-19, Cardiorenal axis, Cardiorenal diseases, Inflammation, Immune system

## Abstract

Recent findings have confirmed relationships between coronavirus disease (COVID-19) and multiple organ dysfunction. The prevalence of cardiac and renal involvement in COVID-19 has been increasingly reported and is a marker of severe disease that not only directly or indirectly affects the organs, but may also exacerbate the underlying comorbid illness. In addition, patients affected by the new coronavirus present a systemic inflammatory condition that results in damage to several tissues, especially the heart, kidneys, and vessels. It is well known that the heart and kidneys are closely related, so that any change in one of the organs can lead to damage to the other, establishing the so-called cardiorenal syndrome. Herein, we explore some case reports of patients with COVID-19 who had heart and kidney abnormalities, consequently resulting in worse prognosis of the disease. These results highlight the importance of understanding the cause and effect between the cardiac and renal systems and the course of early SARS-CoV-2 infection.

## Introduction

Coronavirus disease 2019 (COVID-19) is caused by severe acute respiratory syndrome coronavirus 2 (SARS-CoV-2) and was first reported in December 2019 in the city of Wuhan, China. The disease has spread rapidly around the world, causing about 270 million confirmed cases and more than 5.3 million deaths reported to WHO by December 2021. In addition, there is evidence that some variants and emerging mutations of SARS-CoV-2 can evade immune responses triggered by vaccines and previous infections (1).

The three countries with the highest reported case numbers are the United States, India, and Brazil, with approximately 49, 34, and 22 million confirmed cases, respectively. These countries also have the highest number of deaths, with the United States leading the rank with more than 792,000 deaths. Although India is more than 6 times as populous as Brazil and has more cases overall, Brazil has the second highest number with more than 616,000 deaths, followed by India with 476,000 deaths (2).

Human coronaviruses were identified in 1960 and known to cause only mild respiratory infections. However, they were studied more intensively after the emergence of acute respiratory syndrome coronavirus (SARS-CoV), which caused epidemics in 2002 and 2003, and the Middle East respiratory syndrome virus (MERS-CoV) in 2012. The SARS-CoV-2 virus, the cause of the current COVID-19 pandemic, is the seventh identified human coronavirus and is transmitted from an infected person through saliva droplets and close personal contact (3). The clinical spectrum of COVID-19 ranges from asymptomatic state to severe bilateral or diffuse pneumonia that can lead to acute respiratory distress syndrome (ARDS), respiratory failure, and/or multiple organ dysfunction.

SARS-CoV-2 is a positive-sense single-stranded genomic RNA enveloped virus (+ssRNA). It is considered to belong to the coronavirus family and Sarbecovirus subgenus. According to the genome sequencing, the complete genome of SARS-CoV-2 (4) is around 30 kb and two-thirds contains ORF1ab encoding orf1ab polyproteins, involved in virus transcription and replication, while the other part presents genes encoding structural proteins, M, N, E, and S proteins, known as membrane, nucleocapsid, envelope, and surface glycoprotein, respectively (5).

SARS-CoV-2 enters the host by binding to angiotensin-2 converting enzyme (ACE2), which functions as a receptor for coronaviruses. ACE2 has been characterized since 2000 with a structural genomic sequence similar to the human ACE gene. However, ACE2 has different biological activities from ACE, converting angiotensin 1 (Ang 1) into Ang-(1-9), which is further hydrolyzed by ACE into Ang-(1-7), a vasodilator molecule with cardiovascular effects contrary to Ang 2, which is hypertensive. In addition, ACE2 acts in the process of formation of Ang-(1-7) from Ang 2. Therefore, ACE2, in addition to being a key enzyme in the generation of the potent vasodilator Ang-(1-7), reducing sodium and water retention with a hypotensive effect, is essential for adequate myocardial function and may be cardioprotective (6,7).

The enzyme binds to the surface glycoprotein S, which then triggers cellular response and modulates cellular function. Next, there is proteolytic cleavage of protein S and a protease, which allows fusion in cells called transmembrane serine 2 protease (TMPRSS2), responsible for the reaction and virus entry into cells (5). ACE2 also plays a role in lung protection and therefore viral binding to this receptor disrupts a lung protection pathway. ACE2 is expressed in lungs, heart, intestine, and kidney, justifying the systemic manifestations of COVID-19.

Despite the increasing recovery of patients affected by COVID-19, there are reports of persistence of disease symptoms more than a month after the onset of symptoms and in which the replicating virus was not isolated (8). Therefore, in addition to preventive care against COVID-19, efforts should be made to manage patients affected by the disease and provide multidisciplinary care for survivors at high risk for post-acute (long) COVID-19 syndrome.

## Search strategy

This narrative review captured a subset of recent reports of complications caused by the current new coronavirus pandemic. First, we focused on describing evidence of the association between viral infection and cardiorenal syndrome. The search was carried out on databases covering specific geographic regions from 2019 to 2021 and included index terms and keywords: COVID-19 and cardiorenal syndrome/cardiorenal complications/cardiovascular and renal systems. MEDLINE/PubMed and WHO were searched with the keywords using the standard recommendations for a biomedical review. Next, we highlighted advances in complications of the cardiac and renal systems and discussed how this can be applied to specific clinical situations. For this, relevant articles and their bibliographies were selected and explored.

## COVID-19 and cardiovascular system

SARS-CoV-2 infection generates a variety of clinical manifestations, including asymptomatic cases and rapid deaths (9). Severe systemic symptoms caused by COVID-19 are associated with an enormous inflammatory response, in addition to overproduction of inflammatory cytokines leading to systemic inflammation and multiple organ dysfunction syndrome that acutely affects the cardiovascular system and directly correlates with an unfavorable prognosis (10). In addition, patients infected with SARS-Cov-2 virus have been reported to be at increased risk of developing arterial and venous thromboembolic complications.

Individuals infected with the new coronavirus can present coagulation disorders, increasing the risk of thrombosis and consequently presenting a higher incidence of pulmonary embolism (11). Although the mechanisms involved with thrombosis are still not completely elucidated, the activation of blood coagulation in most patients affected by COVID-19 is caused by high levels of fibrinogen and progressive elevation of D-dimers. In addition, there is release of PAI-1, a procoagulant inhibitor, and endothelial dysfunction, factors that also significantly contribute to thrombogenesis in patients with COVID-19 (12). Furthermore, intense endothelial inflammation was observed in infected patients with very high levels of von Willebrand Factor (vWF), Ag, and FVIII. vWF is a complex plasma glycoprotein that has binding sites to collagen and to coagulation factor VIII (FVIII), justifying its important role in the hemostatic system. Its main functions are platelet adhesion to the exposed collagen of the subendothelium after vascular injury and binding with FVIII, preventing its proteolysis and promoting its stabilization in plasma. Thus, in addition to the increased activation of blood coagulation, low oxygen levels in the lung capillaries can aggravate vascular constriction (13). Therefore, it is important to study cardiovascular complications in order to contribute significantly to the mortality associated with diseases.

SARS-CoV-2 infection occurs through the coupling of ACE2 with ACE2 receptor, which acts as a receptor for the virus. In addition, ACE2 is widely expressed in the heart and kidneys, evidencing the link between the cardiovascular and renal systems with coronavirus infection. ACE2 is down-regulated as the infection progresses, resulting in increased action of angiotensin II and/or loss of cardioprotective effects of angiotensins. Given the relatively high density of ACE2 receptors expressed in cardiomyocytes, SARS-Cov-2 infection may anticipate myocarditis and cause cardiac damage and dysfunction. For this reason, countless studies in the scientific community worldwide have been dedicated to the interactions of SARS-CoV-2 with cardiovascular alterations, since patients affected by the coronavirus present an important systemic inflammatory condition that causes damage to several tissues, especially the heart, kidneys, and vessels (14).

Patients with cardiovascular complications during acute COVID-19 infection may present persistent cardiac symptoms including chest pain, dyspnea, and palpitations. In addition, long-term sequelae may include increased cardiometabolic demand, arrhythmias, tachycardia, and myocardial fibrosis, among others (15). Conditions such as thromboembolism and vascular endothelial damage are subsequent problems after coronavirus proliferation, which can lead to consequences such as stroke, ischemic heart disease, and non-obstructive coronary heart disease, suggesting that the SARS-Cov-2 virus possibly interacts with the cardiovascular system through multiple mechanisms that are not yet fully understood.

Considering that the heart and kidneys have a close functional relationship, it is expected that cardiac alterations observed in COVID-19 are accompanied by renal alterations and vice-versa. Thus, cardiorenal syndrome (CRS) should definitely be studied to better understand the cellular and molecular mechanisms involved in pathologies derived from SARS-CoV-2.

## Impact of COVID-19 on the cardiorenal axis

CRS is characterized by a systemic inflammatory process in which different clinical conditions lead to cardiac and renal dysfunction. CRS has five different types and is divided into 2 main large groups, cardiorenal and reno-cardiac, which can be acute or chronic. Type 1 and 2 CRSs are associated with abnormalities in heart function, which cause kidney damage and/or dysfunction, and types 3 and 4 are characterized by kidney problems that lead to heart dysfunction. Type 5 CRS is characterized by systemic diseases that induce both cardiac and renal dysfunctions (16). The mechanisms that lead to reno-cardiac syndrome include cellular injury-associated chemokine and cytokine secretion, especially interferon-γ, tumor necrosis factor, and interleukin (IL) 1β, with myocardial inflammation, injury, apoptosis, and necrosis. Furthermore, deleterious effects in cardiac electrical activity also occur after acute or chronic kidney injury (17). Activation of the sympathetic nervous system and renin-angiotensin-aldosterone system also contributes to myocardial injury (18).

The renin-angiotensin system plays a central role in cardiovascular physiology and regulation. Many of its effects are modulated by the angiotensin-converting enzyme (ACE), which, when removing two amino acids from Ang 1, forms Ang 2, the most potent vasoconstrictor among those involved in the pathophysiology of arterial hypertension. As mentioned before, Ang 2 can be converted into Ang(1-7) through the action of ACE2. This is important because while Ang 2 binds to its type 1 receptor (AT1) and promotes several inflammatory effects and tissue damage, when Ang (1-7), in turn, binds to its MAS receptor, it promotes opposite effects, i.e., anti-inflammatory, antifibrogenic, vasodilator, cardioprotective, and nephroprotective. Thus, by reducing ACE2 expression, a phenomenon that occurs after the virus binds to the enzyme, there is less conversion of Ang 2 to Ang(1-7) and, consequently, less protective mechanisms and a greater amount of Ang 2, which can worsen the acute lung injury and cause adverse effects on the kidney and heart. Among the symptoms caused by SARS-CoV-2 infection, coagulation disorders, inflammation, and excessive immune response impair the pulmonary, renal, and cardiac physiology, leading to damage to these organs, possibly due to the involvement of the reduction of ACE2 (14).

**Figure 1:**
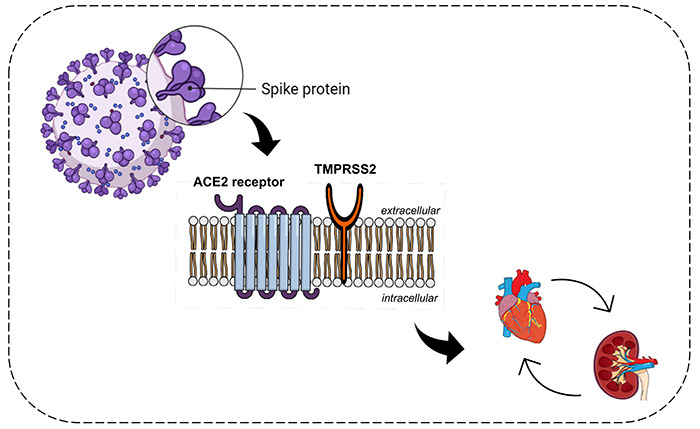
Schematic illustration representing the coronavirus entry into the cell and the consequences involving the cardiorenal syndrome. The spike protein (S) of SARS-CoV-2 allows entry of the virus into cells by binding to the angiotensin-2 converting enzyme (ACE2) and cell fusion provided by the serine protease TMPRSS2 for protein S. ACE2 is expressed in different organs, including the heart and kidneys, which have a close functional relationship in inflammation, electrical mechanisms, and/or activation of the sympathetic nervous system. This characterizes cardiorenal syndrome, in which cardiac and renal dysfunctions overlap. SARS-CoV-2 provides a link between coronavirus infection and cardiovascular and renal alterations.

In view of the above, it is clear that the relationship between the heart and the kidneys is broad and permeates numerous cellular, molecular, and physiological processes. Therefore, the integrated study of these organs may provide more complex and complementary information that can be used as a guide for future medical therapies for disorders affecting at least one of these organs, as is the case with COVID-19. Organ involvement in patients with COVID-19 is well-known, and researchers have recently focused on studying the involvement of kidneys and their interaction with the cardiovascular system (Figure 1).

COVID-19 has been commonly associated with kidney damage, as the proximal tubules contain cells that express ACE2, the receptor for SARS-CoV-2, in large amounts. Coronavirus invasion of renal tissue was demonstrated in a study of 26 Chinese patients in which acute tubular injury was shown in all subjects (19). In addition to the direct viral symptoms associated with SARS-CoV-2, other secondary insults, especially hypoxia, cytokine storms, secondary infections, and drug-associated nephrotoxicity, can contribute to acute kidney injury (AKI) and possibly to future cardiovascular events, such as the higher mortality rate in patients with COVID-19 with AKI compared to those without the AKI.

Scientists suggest that potential causes of COVID-19-associated kidney damage may be explained by direct effects of COVID-19 and indirect effects of systemic inflammation, as well as organ-organ crosstalk. It appears that COVID-19 virus can invade renal cells and lead to clinical manifestations ranging from proteinuria to AKI since SARS-CoV-2 virus has been detected in urine smears from patients with COVID-19 (20).

In a previous study, the kidneys of patients affected by COVID-19 had advanced tubular damage, which showed that SARS-CoV-2 can specifically cause dysfunction in proximal tubules. Evidence of proximal tubule dysfunction in patients infected with SARS-Cov-2 was supported by low molecular weight proteinuria, hyperuricemia, and aminoaciduria. Furthermore, the amounts of uric acid eliminated in excess through the urine was associated with the severity and final outcome of the disease (21).

Initial data from Wuhan showed an important association of patients with COVID-19 and cardiac complications with mortality. Later, a study showed that the cause of death of people who died from COVID-19 was respiratory failure and/or myocardial injury in more than 30% of patients, and the cause in 7% of them was heart failure (21). These observations were corroborated by a study that showed higher rates of ventilation requirement and hospital mortality throughout the disease course in patients with cardiac damage, leading to a higher risk of death (22). Acute myocardial injury is described as the most common cardiovascular complication of COVID-19, and regardless of the incidence in patients infected with SARS-Cov-2, there is likely a development of complications that may increase arrhythmias and sudden cardiac death, for example. The first case study from Wuhan describing the clinical profile of Chinese patients hospitalized with COVID-19 showed that cardiac arrhythmia occurred in almost half of the patients. Furthermore, severe systemic inflammation increases the risk of acute myocardial infarction, so that this risk is likely in patients with severe COVID-19 (23).

Cardiac complications in COVID-19 are mostly associated with a poor clinical outcome. In recent studies, almost 20% of hospitalized patients with COVID-19 had abnormally high levels of troponin I, a cardiac biomarker. Elevated troponin levels are associated with malignant arrhythmias and fatal outcome, and patients with elevated troponin levels had a mortality rate above 50% (22).

Patients who recovered from COVID-19 can have a persistent increase in cardiometabolic demand, which is possibly related to the dysregulation of the renin-angiotensin-aldosterone system, reduced cardiac reserve, and dysregulation of systemic inflammation due to cytokines such as IL-6, IL-1β, and tumor necrosis factor (8). The decompensated heart failure leads to kidney injury, mostly generated by dysfunction of hemodynamic mechanisms, which in turn leads to CRS. Acute or chronic abnormalities in cardiac function lead to dysfunction and decreased renal function due to a lower renal arterial flow and consequent decrease in glomerular filtration rate.

**Figure 2:**
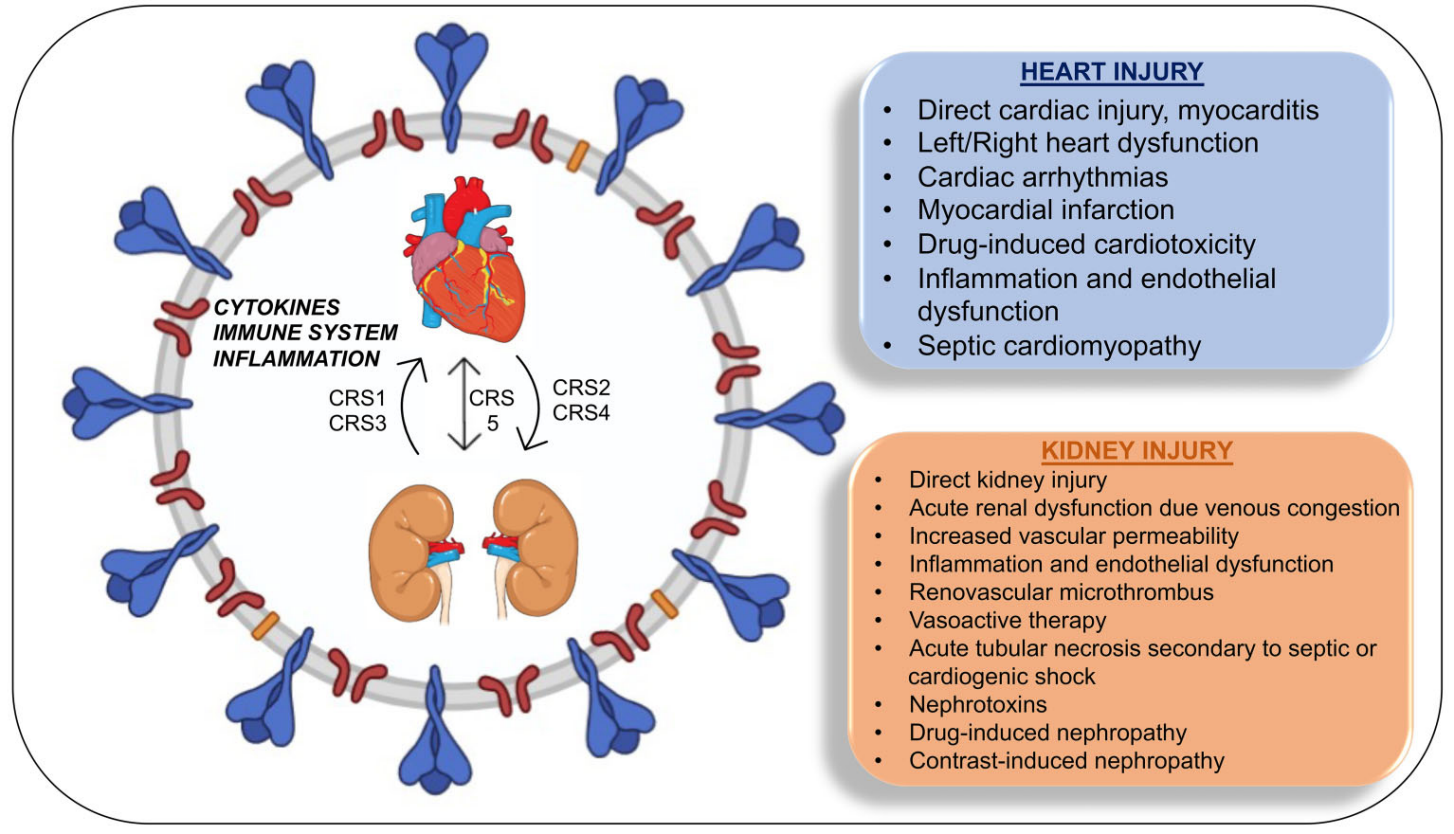
Schematic representation of COVID-19 pathophysiology and its effects on kidney and heart. CRS: cardiorenal syndrome; CRS 1 to 5: types of CRS.

Organs such as the heart and kidneys have a reciprocal relationship, so that acute or chronic dysfunction in one will affect the other. However, the unique changes caused by COVID-19 characterizes the complex pathophysiology basis of COVID-19-related organ abnormalities. SARS-CoV-2 causes renal and cardiovascular complications and the concomitant development of organ damage, such as kidney injury or acute myocarditis, is associated with significantly worse outcomes (Figure 2). Furthermore, the long-term effects of COVID-19-associated AKI and cardiac problems have not been defined; however, established kidney and heart diseases are a risk factor for poor recovery.

## Future perspectives

SARS-CoV-2 infection can be asymptomatic or trigger different signs and symptoms ranging from mild to serious problems such as the overproduction of inflammatory cytokines, which can lead to death. This depends on the interaction of the virus with the host's immune response to COVID-19. Given this, cytokine storms are associated with severe inflammation and injury of vital organs. It remains unclear how COVID-19-induced cytokine signaling occurs at the cellular and molecular levels, since there is no complete elucidation of the inflammatory pathways that define the course of the disease. Therefore, a detailed study of biological interactions in patients affected by SARS-Cov-2 is essential.

Moreover, studies showing the connection between organs affected by COVID-19 are sorely needed, especially those directly related to the cause of death, such as a detailed study of the cellular mechanisms involving kidneys and the heart. Understanding the cellular and molecular mechanisms involved in the pathology and clinical variations of COVID-19 may provide the basis for targeting studies and possible treatments. Finally, both COVID-19 itself and related diseases must be continuously monitored to minimize the likelihood of life-threatening events.

In this scenario, antithrombotic drugs have promising therapeutic mechanisms. RAAS inhibitors can reduce thrombosis. For example, losartan, an angiotensin receptor blocker, and ramipril, an angiotensin-converting enzyme inhibitor, reduce the formation of blood clots in the arteries. Furthermore, amplifying ACE2 function can also have antithrombotic effects, such as the use of ACE1 inhibitors and angiotensin II receptor blockers (ARBs) that have decreased mortality rates in patients with COVID-19. In addition, there are studies showing beneficial effects of drugs with immunosuppression characteristics and mechanisms, since the progression of the disease is related to a severe inflammatory condition (12). Thus, strategies such as antithrombotics and suppressive therapies can enhance other therapies and improve symptoms in patients with COVID-19.

These scientific studies can support new research aimed at finding treatments capable of preventing more serious problems in the systems affected by COVID-19, especially the renal and cardiovascular systems, which are intrinsically related to each other. As new discoveries are disseminated in the scientific community, new mechanisms and interactions are also proposed. Thus, an integrated view between organs could lead to better theranostics for patients affected by COVID 19.

## References

[1] Callaway E (2021). Fast-spreading COVID variant can elude immune
responses. Nature.

[2] World Health Organization (February 15, 2022). WHO Coronavirus (COVID-19) Dashboard: Data information.

[3] Yüce M, Filiztekin E, Özkaya KG (2021). COVID-19 diagnosis-a review of current methods. Biosens Bioelectron.

[4] Wu F, Zhao S, Yu B, Chen YM, Wang W, Song ZG (2020). Author correction: a new coronavirus associated with human
respiratory disease in China. Nature.

[5] Khailany RA, Safdar M, Ozaslan M (2020). Genomic characterization of a novel SARS-CoV-2. Gene Rep.

[6] Rice GI, Thomas DA, Grant PJ, Turner AJ, Hooper NM (2004). Evaluation of angiotensin-converting enzyme (ACE), its homologue
ACE2 and neprilysin in angiotensin peptide metabolism. Biochem J.

[7] Vickers C, Hales P, Kaushik V, Dick L, Gavin J, Tang J (2002). Hydrolysis of biological peptides by human angiotensin-converting
enzyme-related carboxypeptidase. J Biol Chem.

[8] Moreno-Pérez O, Merino E, Leon-Ramirez JM, Andres M, Ramos JM, Arenas-Jiménez J (2021). Post-acute COVID-19 syndrome. Incidence and risk factors: a
Mediterranean cohort study. J Infect.

[9] Petersen E, Koopmans M, Go U, Hamer DH, Petrosillo N, Castelli F (2020). Comparing SARS-CoV-2 with SARS-CoV and influenza
pandemics. Lancet Infect Dis.

[10] Mehta P, McAuley DF, Brown M, Sanchez E, Tattersall RS, Manson JJ (2020). COVID-19: consider cytokine storm syndromes and
immunosuppression. Lancet.

[11] Ahmed S, Zimba O, Gasparyan AY (2021). COVID-19 and the clinical course of rheumatic
manifestations. Clin Rheumatol.

[12] Ahmed S, Zimba O, Gasparyan AY (2020). Thrombosis in coronavirus disease 2019 (COVID-19) through the
prism of Virchow's triad. Clin Rheumatol.

[13] Helms J, Tacquard C, Severac F, Leonard-Lorant I, Ohana M, Delabranche X (2020). High risk of thrombosis in patients with severe SARS-CoV-2
infection: a multicenter prospective cohort study. Intensive Care Med.

[14] Cruz NAN, de Oliveira LCG, Silva HT, Pestana JOM, Casarini DE (2021). Angiotensin-converting enzyme 2 in the pathogenesis of renal
abnormalities observed in COVID-19 patients. Front Physiol.

[15] Ronco C (2010). Cardiorenal syndromes: definition and
classification. Contrib Nephrol.

[16] Trentin-Sonoda M, da Silva RC, Kmit FV, Abrahão MV, Monnerat Cahli G, Brasil GV (2015). Knockout of toll-like receptors 2 and 4 prevents renal
ischemia-reperfusion-induced cardiac hypertrophy in mice. PLoS One.

[17] Panico K, Abrahão MV, Trentin-Sonoda M, Muzi-Filho H, Vieyra A, Carneiro-Ramos MS (2019). Cardiac inflammation after ischemia-reperfusion of the kidney:
role of the sympathetic nervous system and the renin-angiotensin
system. Cell Physiol Biochem.

[18] Su H, Yang M, Wan C, Yi LX, Tang F, Zhu HY (2020). Renal histopathological analysis of 26 postmortem findings of
patients with COVID-19 in China. Kidney Int.

[19] Pan XW, Xu D, Zhang H, Zhou W, Wang LH, Cui XG (2020). Identification of a potential mechanism of acute kidney injury
during the COVID-19 outbreak: a study based on single-cell transcriptome
analysis. Intensive Care Med.

[20] Werion A, Belkhir L, Perrot M, Schmit G, Aydin S, Chen Z (2020). SARS-CoV-2 causes a specific dysfunction of the kidney proximal
tubule. Kidney Int.

[21] Ruan Q, Yang K, Wang W, Jiang L, Song J (2020). Clinical predictors of mortality due to COVID-19 based on an
analysis of data of 150 patients from Wuhan, China. Intensive Care Med.

[22] Shi S, Qin M, Shen B, Cai Y, Liu T, Yang F (2020). Association of cardiac injury with mortality in hospitalized
patients with COVID-19 in Wuhan, China. JAMA Cardiol.

[23] Driggin E, Madhavan MV, Bikdeli B, Chuich T, Laracy J, Biondi-Zoccai G (2020). Cardiovascular considerations for patients, health care workers,
and health systems during the COVID-19 pandemic. J Am Coll Cardiol.

